# Demonstrating the Synthesis and Antibacterial Properties of Nanostructured Silver

**DOI:** 10.1021/acs.jchemed.3c00125

**Published:** 2023-08-16

**Authors:** Lewis Rolband, Varsha Godakhindi, Juan L. Vivero-Escoto, Kirill A. Afonin

**Affiliations:** † Department of Chemistry, University of North Carolina at Charlotte, Charlotte, North Carolina 28223, United States

**Keywords:** Upper-Division Undergraduate Education, Graduate Education, Interdisciplinary, Laboratory Exercises, Biotechnology Education, Nanotechnology, Nucleic Acids/DNA/RNA, Oxidation/Reduction, Silver nanoparticles, Silver nanoclusters

## Abstract

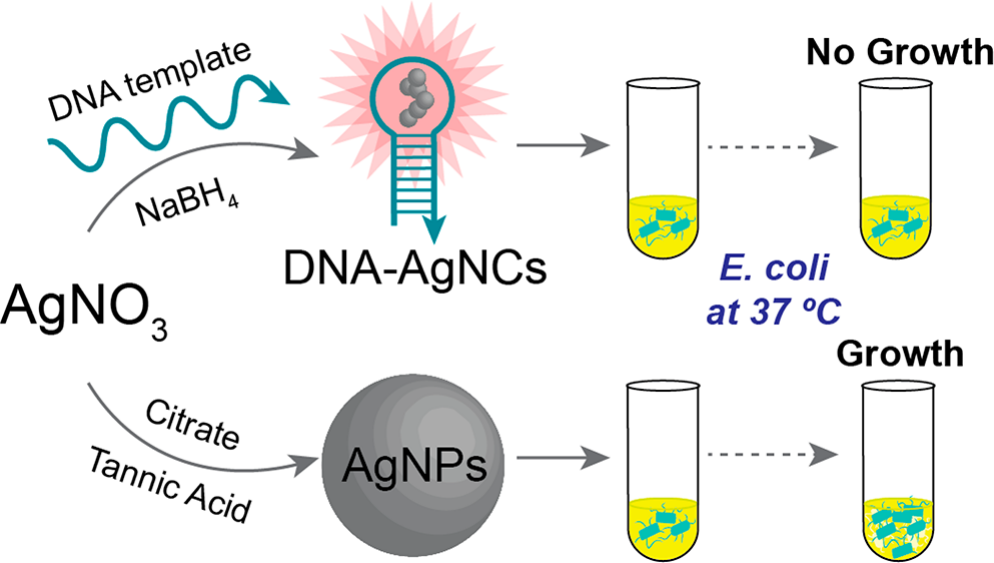

Investigating and understanding novel antibacterial agents is a necessary task as there is a constant increase in the number of multidrug-resistant bacterial species. The use of nanotechnology to combat drug-resistant bacteria is an important research area. The laboratory experiment described herein demonstrates that changes in the nanostructure of a material lead to significantly different antibacterial efficacies. Silver has been known to be an effective antibacterial agent throughout history, but its therapeutic uses are limited when present as either the bulk material or cations in solution. Silver nanoparticles (AgNPs) and DNA-templated silver nanoclusters (DNA-AgNCs) are both nanostructured silver materials that show vastly different antibacterial activities when incubated with *E. coli* in liquid culture. This work aims to provide students with hands-on experience in the synthesis and characterization of nanomaterials and basic microbiology skills; moreover, it is applicable to undergraduate and graduate curricula.

## 1 Introduction

Since the advent of antibiotics in the early 19th century, there has been significant improvement in the effort to combat bacterial infections; however, the increased prevalence of antibiotic resistance has become a significant challenge. The widespread overuse of antibiotics in livestock, agriculture, and medicine, combined with the scarcity of new therapeutics, has led to the emergence of multidrug-resistant (MDR) bacteria.^1^ The surge in MDR bacteria threatens to setback all the previous advances made toward treating bacterial infections. MDR strains of common bacteria also negatively impact the clinical outcome of a wide range of hospital-bound groups, including those in intensive care units, undergoing surgery, transplantation, or cancer treatment.^2^ The WHO Global Antimicrobial Surveillance System’s 2017 report labels antibiotic resistance as a worldwide challenge, with the estimated cost of treating antibiotic-resistant infections being approximately $20 billion annually.^3^

The initial success of antibiotics was attributed to the specificity of their antimicrobial activity, which targeted the bacterial membrane, key proteins, oligonucleotide synthesis, and metabolic pathways. As bacteria possess the intrinsic ability to evolve rapidly through mutations and horizontal gene transfer between bacteria, they are able to rapidly overcome the threat posed by these antibiotics.^4^ As a result, alternative antibacterial agents must be explored to combat antibiotic resistance. Among the possible alternative treatments, nanomaterials have demonstrated promising results through the disruption of the bacterial membrane and the targeting of intracellular and membrane-bound proteins.^2^ By taking advantage of multiple, nonspecific, antibacterial mechanisms, bacteria become less prone to develop resistance against nanoparticles.^5^

Silver, as a cation species (Ag^+^), or as colloidal nanoparticles, has been extensively used for wound treatment and as a biocide. However, comprehensive research detailing the antibacterial mechanism of colloidal silver did not emerge until 2004.^6^ Silver nanoparticles (AgNPs) provide an obvious advantage over other forms of silver, showing antibacterial activity at lower doses that present minimal toxicity to humans while overcoming many antibiotic resistance mechanisms in bacteria. This antibacterial activity relies on the release of Ag^+^ and the ability of Ag^+^ to disrupt key bacterial components such as the cell wall, DNA, and intracellular and membrane-bound proteins.^7,8^ Furthermore, the release of Ag^+^ facilitates the formation of reactive oxygen species which can induce cell death through the increased oxidative stress placed on the bacterial cell.^9^ The antibacterial efficacy of AgNPs is generally limited in liquid medium due to their low colloidal stability, an effect which is clearly demonstrated in the series of experiments presented herein.^10^ Chemical reduction of silver nitrate (AgNO_3_) by organic or inorganic reducing agents is the most common approach to the synthesis of AgNPs. Sodium citrate and tannic acid serve two purposes in this synthesis as they act to reduce Ag^+^ to Ag^0^ and to increase the colloidal stability of the resulting AgNPs.^11^ Tannic acid and sodium citrate form a complex in solution, which then undergoes oxidation to contribute to the reduction of Ag^+^.^11^ While the chemistry of this reaction has yet to be fully elucidated, the trisodium citrate-based reduction can be illustrated by the following chemical reaction:
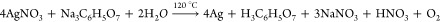


As another class of nanostructured silver, DNA-templated silver nanoclusters (DNA-AgNCs) are now being actively investigated for their antibacterial and biosensing capabilities.^12−19^ Cytosine-rich single-stranded oligonucleotides are most frequently used as a template for DNA-AgNC formation since, of all the nucleobases, cytosines have the highest affinity for Ag^+^.^20,21^ DNA-AgNCs are able to form on and induce the formation of a wide variety of DNA conformations and are stabilized by their DNA template over a wide range of pH and ionic strength conditions.^20,22−25^ The synthesis of DNA-AgNCs is both simple and reliable, requiring only the template oligonucleotide, silver nitrate (AgNO_3_), sodium borohydride (NaBH_4_), and ammonium acetate (NH_4_O_2_C_2_H_3_) solution at pH 6.9. The relevant chemical reaction occurs between AgNO_3_ and NaBH_4_, after the Ag^+^ ions have had an opportunity to bind the template, as follows:
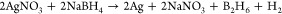


Upon formation, the unique fluorescent properties of DNA-AgNCs become apparent, as the small collection of silver atoms gains molecule-like electronic properties.^26^ Hairpin-DNAs reliably form monodisperse populations with well-defined fluorescence.^27^ Single-stranded DNAs with equivalent numbers of cytosines tend to aggregate and form oligomers bridged by silver atoms when the same synthetic route is applied.^15^ The DNA-AgNCs formed on a DNA hairpin with 13 single-stranded cytosines (DNA(C13)-AgNCs), used in this curriculum as model system, were recently found to be effective against *E. coli* in liquid cultures.^15^

Though DNA(C13)-AgNCs and AgNPs both contain nanostructured silver atoms, their physicochemical and biological properties are significantly different. The differences in the optical properties of DNA(C13)-AgNCs and AgNPs are readily observable. DNA(C13)-AgNCs are known to fluoresce upon absorbing UV or visible light, while AgNPs absorb visible light without demonstrating fluorescence, with the wavelength of peak absorbance depending heavily on the size of the AgNPs through surface plasmon resonance.^13−15,17,22,24,25,28,29^ Both of these nanostructures have shown antibacterial efficacy, but their direct comparisons have been limited. Herein, we report a laboratory curriculum that demonstrates how differences in nanostructures of the same chemical species (Ag) can lead to different antimicrobial performances by allowing students to synthesize and directly compare AgNPs and DNA(C13)-AgNCs. In particular, the lack of colloidal stability of AgNPs in an LB medium renders them ineffective against *E. coli*, while water-soluble DNA(C13)-AgNCs remain in solution for extended periods of time and efficiently inhibit bacterial growth. This curriculum allows students to gain firsthand experience in metal nanoparticle synthesis, synthetic biotechnology, and molecular biology techniques in a safe and effective manner. Additionally, the developed laboratory work allows students the opportunity to perform a series of experiments that (i) replicate the experience of researchers who are actively investigating the antibacterial efficacy of various nanoformulations, (ii) compare side-by-side defifferent silver nanomaterials, and (iii) draw conclusions based on their observations of variances in antibacterial activities.

With the development and implementation of this experimental series, our goals were twofold. First, we aimed to introduce students to the health-relevant problems (bacterial infections) and applied nanochemistry with synthesis and characterization of two separate silver nanomaterials that are being actively studied for their antibacterial properties. As a part of this introduction, we can show students, in easily observable ways, how the differences in the structure of the silver in each nanomaterial greatly impact the optical, antibacterial, and colloidal properties of the resulting formulations. Second, we wished to provide students of different levels of experience and preparations with an experimental toolkit that they can bring forward with them in their careers. In contrast to previous laboratory exercises which involved the use of silver or other nanomaterials as antibacterial agents, the current experiments demonstrate how differences in the nanostructures of two systems made of the same material lead to significantly different physicochemical and biological properties.^30,31^ These experiments teach students how to use common chemical glassware and common biochemistry and microbiology equipment. These experiments also provide students with hands-on experience with the synthetic procedures required for inorganic and bioinorganic hybrid nanomaterials. The concluding experiments in the series also teach basic microbiology procedures and a method for assessing antibacterial activity.

## 2 Experimental Section

### 2.1 Safety

To carry out this experiment safely, the laboratory space should be kept clean and a disinfectant solution (10% bleach) should be readily available to disinfect working areas before and after each laboratory session. Students and instructors should always wear personal protective equipment (PPE), including a laboratory coat, gloves, and UV-protective safety glasses. Heat protective PPE should be used while handling glassware during silver nanoparticle synthesis. All container lids should be tightly secured during centrifugation steps. Flammable liquids should be appropriately stored while Bunsen burners are lit, and lit Bunsen burners should never be left unattended. If available, biosafety cabinets should be used instead of a flame to maintain the aseptic conditions. All participants should wash their hands immediately after removing their PPE once they have finished working with bacteria cultures. While the K-12 strain of *E. coli* is generally considered nonpathogenic, it is a biosafety-1 organism and institutional biosafety committee approval should be obtained. It is imperative that all biological waste is sequestered, autoclaved, or otherwise disposed of in a safe manner. While the bacteria cultures are shaking, their lids should be on but loose to ensure there is no cross-contamination or splashing. Any spills should be cleaned with a 10% bleach solution. All work surfaces should be decontaminated with 10% bleach at the end of each lab period.

The experimental procedures include the use of nanoscale materials. As such, it is recommended by the Occupational Safety and Health Administration (OSHA) to always wear the PPE described above during laboratory work and to wash hands immediately after the completion of all experimental work. Safety procedures should be established by the instructor to clean spills as soon as possible after they occur. The synthesis of AgNPs should be performed in a fume hood. Students should also be trained on the proper pipet technique to minimize the risk of any nanomaterials becoming aerosolized. All nanomaterial containing solutions should be disposed of as hazardous waste, as there is the potential for silver nanomaterials being harmful to the environment at-large.^32^

### 2.2 Hazards

The chemicals used in these experiments are not listed as carcinogens in the National Toxicology Program’s 15th Report on Carcinogens and do not pose any reproductive toxicity. The instructors should take extreme precautions while handling aqua regia (3-parts HCl: 1-part HNO3) for glassware cleaning by wearing gloves that are specifically rated for use with concentrated acids in addition to working with this material in a fume hood and wearing all previously mentioned PPE. Aqua regia should only be handled by the instructor for glassware cleaning. Aqua regia is an extremely corrosive solution that can cause explosions, skin burns, or eye/respiratory tract irritation. Silver nitrate is a potent oxidant that can form explosive mixtures with ammonia and combustibles. Upon skin exposure, silver nitrate solution produces temporary dark-colored stains. Sodium borohydride is a strong reducing agent, poses an inhalation risk, and can irritate the skin and eyes. All work with sodium borohydride solids should take place within a fume hood. In the case of skin contact, skin is rinsed with water/shower and the student should seek medical care. All of the chemicals listed in the manuscript pose an acute hazard for aquatic life and must be disposed of accordingly.

### 2.3 Silver Nanocluster and Nanoparticle Synthesis

**Figure 1:**
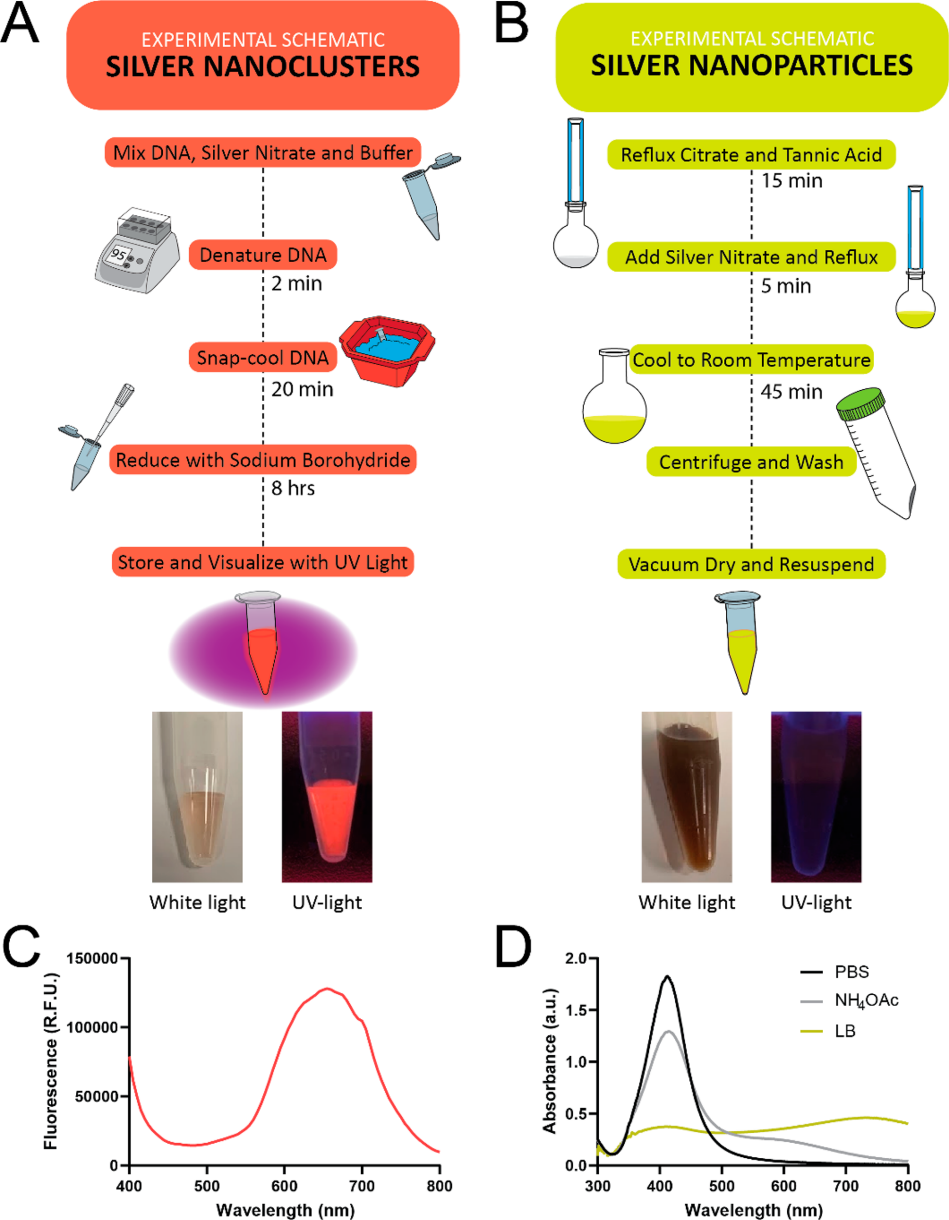
Schematic showing the synthetic route for the formation of (A) fluorescent DNA(C13)-AgNCs and (B) AgNPs. These schemes provide a summary of the experimental steps and can be provided to students to familiarize them with the overall procedure. Pictures of the expected results are shown beneath each scheme under white light and transillumination with a 254 nm UV-light source. (C) The fluorescence spectrum of the DNA(C13)-AgNC is shown with excitation at 260 ± 20 nm. (D) The absorbance spectrum of the AgNPs (15 μg/mL) in phosphate buffered saline (1 mM PBS, black line), 4 mM ammonium acetate (gray line), and Luria broth (LB, yellow line) is shown. The aggregation of AgNPs results in a redshift in the absorbance spectrum.

Synthesizing DNA(C13)-AgNCs (Figure 1A) can be accomplished in a classroom or teaching laboratory setting using well-established protocols.^29^ As the structure of the templating DNA can influence the formation of the DNA(C13)-AgNCs, the hairpin-forming oligonucleotide is first denatured by heating and subsequently folded by cooling in the presence of silver ions and buffer. By rapidly (snap) cooling the solution, the intramolecular hydrogen bonding of the hairpin stem is favored and the oligonucleotide can fold into its intended secondary structure.^15,29,33^ Once the silver cations have bound the templating oligonucleotide, they are reduced with an equimolar amount of sodium borohydride (Figure 1A). There is generally no color change immediately upon the addition of the reducing agent, although the solution may appear to become tinted brown slightly. After being allowed to develop for at least 8 h in the dark, at 4 °C, the fluorescence is readily visualizable upon excitation with ultraviolet (260 nm) light. As a control, a second experiment can be run simultaneously by omitting the templating DNA strand to show that no fluorescent clusters are formed in this case. Upon the addition of sodium borohydride to the control solution, lacking a DNA template, the solution immediately turned dark brown.

The synthesis of AgNPs (Figure 1B) is also a simple procedure that can be demonstrated in a teaching laboratory with standard glassware and minimal equipment. In our approach, sodium citrate acts as both a reducing and stabilizing agent, whereas tannic acid serves as a reducing agent.^34^ Briefly, the aqueous solution of sodium citrate and tannic acid is mixed and brought to a boil under reflux condition. The silver nitrate is added in one portion (Figure 1B), which is a critical step to allow for consistent nucleation. The immediate change in color from a colorless to bright yellow solution indicates the presence of AgNPs. The reaction is removed from heating 5 min after the solution changes color and is allowed to cool to room temperature. The final AgNPs are collected via centrifugation and washed three times with water (Millipore, 18MΩ). The AgNPs obtained are stabilized by the presence of citrate ions on their surface. The successful synthesis of AgNPs was confirmed by an absorbance peak at 420 nm through UV–vis spectrophotometry (Figure 1D).

### 2.4 Bacterial Growth Inhibition Assay

**Figure 2:**
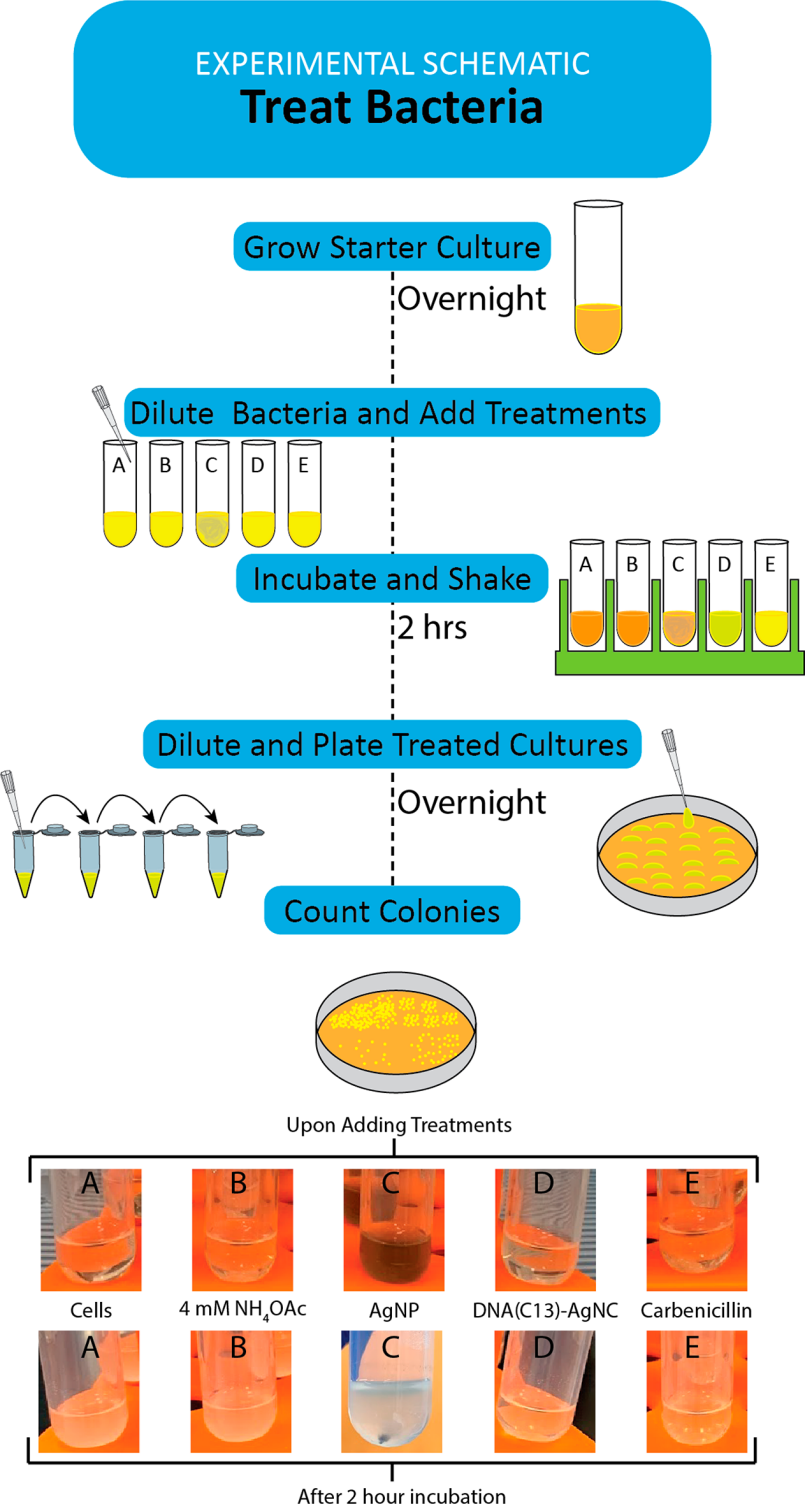
Schematic depicting the treatment, dilution, and plating of the experimental bacterial cultures. The labels on the tubes correspond to the data shown in Figure 3. A color change is observed when AgNPs are added to the bacterial culture, which is illustrated here as a darkening of the solution in tube C. This figure can be provided to students as a roadmap through the key steps of the procedure. Representative photographs of each treatment upon addition to the bacterial culture tubes and after incubation at 37 °C for 2 h with constant shaking at 200 rpm are shown below the scheme. The AgNPs are seen to have fully precipitated in tube C following the incubation period.

Assessing the ability of a material to inhibit the growth of a bacterial species is a key skill in microbiology. As such, many methods have been developed to enumerate the number of viable bacteria in a culture sample, including spread plates, drop plates, and optical density measurements.^35−39^ The K12 strain of *E. coli* used in this work is nonpathogenic, readily available from commercial sources, and grows reliably in a wide array of conditions, making it ideal for use as a safe model system in the classroom.^40,41^ The students mix the treatments they prepared, as well as several controls, and incubate them for 2 h before beginning the process of enumerating the bacteria in the culture. To ensure a fair comparison between AgNPs and DNA(C13)-AgNCs, they are used at equivalent mass concentrations. The drop plate method was chosen in this experiment due to the tendency of AgNPs to aggregate upon addition to the bacterial growth medium and absorb 600 nm light.^42^ The drop-plate method allows for the calculation of colony-forming units (CFU) of bacteria in the culture (Figure 2). After the bacterial cultures have been incubated with the treatments for 2 h, they are serially diluted by factors of 10. Known volumes of each dilution are then placed onto an agar plate with a growth medium. Then, the plates are allowed to dry within the sterile field area near the Bunsen burner or other aseptic laboratory conditions (Figure 2). Following an overnight incubation (12–16 h), the number of colonies present in each drop can then be counted and the colony forming units per milliliter, CFU/mL, in the initial culture can be calculated as shown in eq 1.
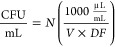
1

**Figure 3:**
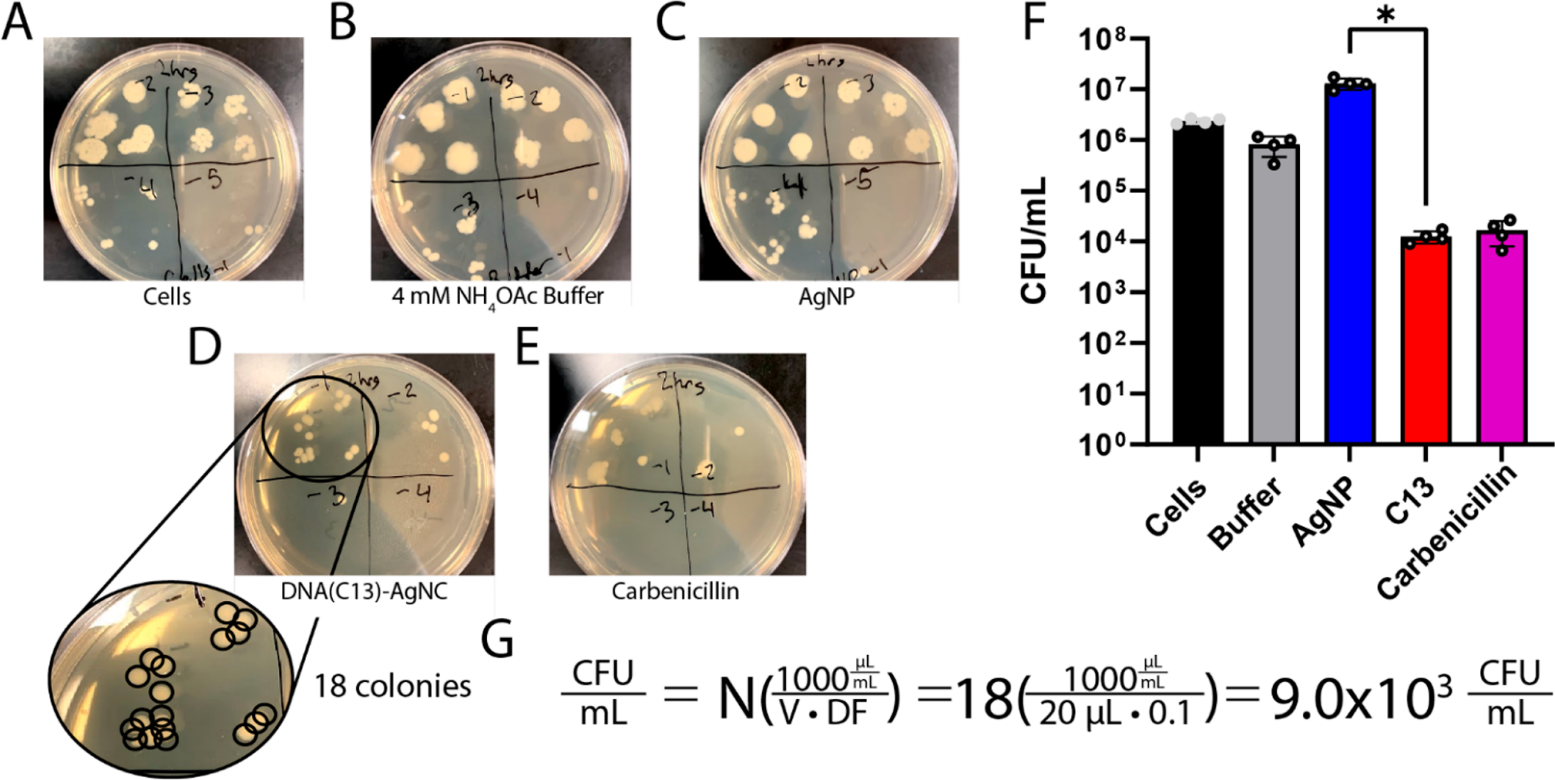
Representative LB-agar plates are shown with the dilution factors written over each sector of the plate for (A) untreated cells, (B) ammonium acetate buffer, (C) AgNPs, (D) DNA(C13)-AgNCs, and (E) carbenicillin after incubation at 37 °C for 2 h with constant shaking at 200 rpm. (F) The instructor’s data from four repeats is shown (mean ± standard deviation) with each individual data point shown as a gray circle. The difference in bacterial growth after 2 h between the DNA(C13)-AgNCs and AgNPs was found to be statistically significant to *p* < 0.01. (G) An example of the calculation is provided for determining the number of colony forming units per milliliter of solution, CFU/mL, in the case of plate D, which shows 18 colonies having been counted, *N*, 20 μL of the bacterial solution having been plated, *V*, from the 10^–1^ (1:10) dilution factor, *DF*.

In eq 1, the concentration of viable bacteria in the liquid culture is given in CFU/mL. The number of colonies, *N*, should ideally be between 20 and 300 in each plate section for reliable counting.^38,39^ The total volume (in microliters) plated in that dilution is given by *V*, and the dilution factor of the counted dilution is indicated by *DF* (Figure 3). The conversion factor from microliters to milliliters is built into this equation.

## 3 Curriculum Design

This experiment was designed to provide students with a broad overview of the synthesis and applications of silver nanotechnologies in a biological context. This series of experiments also mimics our team’s recent research work, allowing students the opportunity to perform this work in a manner that recreates a research laboratory environment.^15^ The developed hands-on activities provide students with the opportunity to safely synthesize AgNPs using standard chemical glassware and methods, produce DNA(C13)-AgNCs using standard biochemistry equipment and techniques, and assess bacterial growth inhibition using introductory microbiology assays. The differences in optical properties of silver nanomaterials can be easily visualized by students and allow them to quickly recognize the effect that the nanoscale structure of the materials has on their functional properties. The relative changes in growth of the bacterial cultures upon treatments with different nanomaterials can be readily assessed by the naked eye, allowing the students to easily observe the differences in the efficacy of each treatment. The application of the drop plate method further allows for these differences to be described quantitatively. By the end of this experimental series, students gain a greater appreciation and understanding of how nanostructural differences in the same chemical compound, silver in this case, lead to different functional properties through their fluorescence, visible light absorbance, colloidal stability (as seen by the aggregation of the AgNPs in the bacterial growth media), and antibacterial activity.

The described procedures are recommended to take place over a total of 4 days. Each step can be easily performed within a 3 h laboratory period. The AgNP synthesis is recommended to be performed first. AgNPs should be stored at room temperature and in the dark. DNA(C13)-AgNCs should be made no more than 1 week before the antibacterial assays are carried out, as the fluorescence and antibacterial activity of clusters can change after long-term storage. DNA(C13)-AgNCs should be stored at 4 °C away from light (e.g., tubes with DNA(C13)-AgNCs can be wrapped in aluminum foil).^15^ Ideally, the DNA(C13)-AgNCs should be made the day prior to the antibacterial assays. To encourage students to test their own hypotheses on what may affect the efficacy of the treatments (concentration, synergistic effects, pH, etc.), students may make additional samples as part of this experimental series or as part of an independent follow-up project.

Most of the preparations for this experimental series can be done well in advance by the instructor. These include the growth of starter *E. coli* cultures prior to the third day of the experiment, the preparation of all stock solutions, and the preparation of LB agar plates. Additionally, instructors should take great care to watch students use all equipment, such as centrifuges and Bunsen burners, to ensure that they are being used safely and effectively. All the reagents are relatively inexpensive and, except for sodium borohydride and the DNA template, can be stored at room temperature with long-term stability. The sodium borohydride solution should be prepared fresh by using cold water and stored in an ice-bath until use. DNA templates should be stored frozen until ready for use and kept at 4 °C or on ice when in use. Student guides, which may be used as handouts, are provided in the Supporting Information that includes the concentration of stock solutions, volumes to be added at each step, and detailed protocols. The instructions required to prepare the stock solutions and bacterial cultures are listed in the instructor guide. During the laboratory periods, students are encouraged to work in groups, although they may work separately if there is enough room and material. While working in groups, students are encouraged to divide labor to perform different portions of each experiment so that they all can receive a comparable experience. For example, one student would make the DNA(C13)-AgNCs while another student in the group made the control solution without a DNA template.

This experimental series was offered to students as part of a week-long silver nanotechnology workshop in the summers of 2021 and 2023, hosted by the authors at the University of North Carolina at Charlotte. It was also offered as part of a doctoral-level nanomedicine course over 3 days in 2022. Overall, ∼ 30 students completed the experiments while working in groups of 2 and/or 3. Both group sizes were used, and the experiments were able to be completed in the same amount of time. These experiments can be adapted to fit the weekly laboratory period model. To do so, the AgNPs would be made in the first week and stored away from light at room temperature. For the second week, DNA(C13)-AgNCs would be made and stored at 4 °C in the dark. The third week would include treatment of the bacteria. The day following this experiment, the instructor would need to collect each group’s colony counting data for them and send it to each group.

This curriculum is designed to use materials and instruments that are typical of an undergraduate chemistry or microbiology laboratory, such as incubators, standard glassware, and spectrophotometers. Students were asked to take a voluntary survey following completion of this course. The students were all from biology or chemistry undergraduate backgrounds and ranged from rising juniors to third-year Ph.D. students enrolled in the Nanoscale Science Ph.D. program at the University of North Carolina at Charlotte. All responders reported having gained a deeper understanding of nanomaterials and methods for testing antibacterial materials. They also self-reported that they learned new synthetic laboratory techniques as a direct result of this experience. Students all stated that following the conclusion of the course, they felt as though they had a working knowledge of basic microbiology laboratory techniques and of the synthesis, optical properties, and biological activities of the two silver nanomaterials that were studied, indicating that the major learning objectives of the course were met. A short quiz was also implemented at the end of the summer 2023 course as an objective means of ensuring that the learning objectives were met. The average score was ∼85%, further indicating that the students had met the learning objectives of the course. Despite the wide variety of student experience levels, this experimental series provided a unique learning experience and deepened students’ understanding of the synthesis, characterization, and biological applications of silver nanotechnologies.

## 4 Benefits of the Approach

This is a relatively inexpensive series of experiments that can be performed with instrumentation that is generally expected to be found in standard introductory chemistry and biochemistry laboratories. All of the main conclusions are easily observed with the naked eye and supported by the data that students collect. The visualization of fluorescent DNA(C13)-AgNCs under UV light and the absorbance of 420 nm light from the AgNPs, as well as their distinct yellow color in solution, jumpstart the conversation of how a material’s nanostructure can change its optical properties. Demonstrating a potential application of these materials as antibacterial agents fosters this discussion by showcasing the application of these nanomaterials in a biological context using a nonpathogenic model system. All of the reagents and materials are of relatively low cost and are readily available from a variety of commercial suppliers. As such, these experiments are expected to be accessible to a wide range of students at the undergraduate and graduate levels. During this course, students learn several essential lab techniques and work with a variety of chemical (e.g., pH meters, thermometers, balances), biochemical (e.g., centrifuges, imagers), and biophysical (e.g., UV–vis and fluorescent spectrophotometers) instruments, providing tremendous assets in their career progression and additional hands on research experience in the healthcare related field of study.

## 5 Conclusion

In this newly developed course, students are provided with the opportunity to study innovative research in therapeutic and nucleic acid nanotechnology and learn to synthesize and characterize nanostructures that carry certain functions designed to impact our lives. By performing this series of experiments, students can interact with nanomaterials first-hand and see how differences in nanostructure can greatly alter the function of a chemical species. In the case of DNA(C13)-AgNCs versus AgNPs, this is clearly shown by the differences in their optical properties, colloidal stabilities, and antibacterial efficacies. Both technologies are being actively researched for antibacterial, among many other, applications. This experimental series provides context for this ongoing research and highlights its biological relevance for students.

## Supporting Information Available

AgCtrl samples and the averaged data from five groups of students (PDF, DOCX)
Instructor Guide (PDF, DOCX)
Student Guide (PDF, DOCX)
