# Supplementary material for: Demonstrating the Synthesis and Antibacterial Properties of Nanostructured Silver
Source: J Chem Educ. Author manuscript; Available in PMC 2023 Sep 19. (PMC10501122; doi:10.1021/acs.jchemed.3c00125)
Supplement: SI4 [file NIHMS1930025-supplement-SI4.docx]

Supporting Information

**Demonstrating the Synthesis and Antibacterial Properties of Nanostructured Silver**

Lewis Rolband^1#^, Varsha Godakhindi^1#^, Juan L. Vivero-Escoto^1*^, Kirill Afonin^1*^

^1^Department of Chemistry, University of North Carolina at Charlotte, Charlotte, North Carolina 28223, United States

^#^L.R. and V.G. contributed equally

^*^Corresponding Authors: kafonin@uncc.edu; [juan.vivero-escoto@uncc.edu](mailto:juan.vivero-escoto@uncc.edu)

**Table of Contents:**

1. **Silver Nanoparticles (AgNPs) Synthesis**

**Timetable**

**Preparation of Materials**

Solutions and Glassware cleaning

**Protocols**

AgNP synthesis and Characterization

**Sample Pre-Lab Questions**

**Sample Post-Lab Questions**

1. **DNA-templated Silver Nanoclusters (DNA(C13)-AgNCs) Synthesis**

**Timetable**

**Preparation of Materials**

Buffers and Solutions

**Protocols**

DNA Resuspension

Synthesis and Visualization

**Sample Pre-Lab Questions**

**Sample Post-Lab Questions**

1. **Antibacterial Efficacy Experiments**

**Timetable**

**Preparation of Materials**

Lysogeny Broth (LB)

Pour LB agar plates

Plate K12 *E. coli*

**Protocols**

Starter Culture Preparation

Dilution and Treatment of Bacteria

Serial Dilution and Plating Treated Bacteria

**Sample Pre-Lab Questions**

**Sample Post-Lab Questions**

1. **Reagents Cost Estimate**
2. **References**
3. **Silver Nanoparticle Synthesis**

| **Timetable** |  |
| --- | --- |
| Glassware Cleaning | 30 minutes |
| Nanoparticle Synthesis | 1 hour |
| Purification and Characterization | 3 hours |

| **Preparation of Materials** | |
| --- | --- |
|  | |
| **Solutions and Glassware cleaning**  *^*Prepare prior to the class session^* | |
|  | **Materials**   - Nitric Acid (HNO_3_) CAS: 7697-37-2 - Hydrochloric Acid (HCl) CAS: 7647-01-0 - Double-deionized water (ddiH_2_O), 18 mΩ CAS: 7732-18-5 - Trisodium citrate CAS: 68-04-2 - Tannic acid CAS: 1401-55-4 - Silver Nitrate (AgNO_3_), 25 mM CAS: 7761-88-8   **Equipment**   - Chemical Fume hood - Round bottom flask [250 mL] - Stir bar - Beaker - Graduated Cylinder [25 mL] - Glass Pipette - Beakers - Graduated cylinder - Precision balance - Weighing paper - Scoopula - Micropipette and pipette tips (1000 µL) - Sharpie marker - Glass vial (20 mL) |
|  | Preparation of Aqua Regia   1. Handle all the chemicals used in this and the following step under a chemical fume hood and follow the relevant safety protocols. 2. Using a graduated cylinder, measure the relevant volume of HCl and HNO_3_ such that their volumetric ratio is 3:1. (*e.g.,* Mix 30 mL of HCl and 10 mL of HNO_3_ to get 40 mL of aqua regia). 3. Mix the measured volumes of HCl and HNO_3_ in a beaker. This solution should appear yellow.   Cleaning the Round bottom flask   1. Place the stir bar in the round bottom flask. 2. To this, add the previously prepared aqua regia. 3. Carefully, swirl the solution inside the flask to allow maximal coating. 4. Repeat the previous step for at least 5 minutes. 5. Discard the solution and rinse the glassware and stir bar with cold ddiH_2_O. 6. Allow the glassware and the stir bar to dry before synthesis.   Preparation of 25 mM of silver nitrate- 10 mL   1. Take a fresh scintillation vial and weigh 43 mg of silver nitrate (AgNO_3_). 2. Add 10 mL of ddiH_2_O and dissolve the contents using a vortexer. 3. Store the prepared solution at room temperature, covered in foil for use up to a month. |
| **AgNP Synthesis and Characterization**  ^*Suggested to be done with students^ | |
|  | **Materials**   - ddiH_2_O CAS: 7732-18-5 - Trisodium citrate CAS: 68-04-2 - Tannic acid CAS: 1401-55-4 - Silver Nitrate (AgNO_3_), 25 mM CAS: 7761-88-8 - Quartz cuvette - Centrifuge tube (50 mL)   **Equipment**   - Cleaned Round bottom flask (250 mL) - Cleaned Stir bar - Beakers - Graduated cylinder - Oil bath - Hot plate with stirrer - Temperature probe - Condenser - Precision balance - Weighing paper - Scoopula - Micropipette and pipette tips (1000 µL) - Sharpie marker - Glass vial (20 mL) - Centrifuge - UV-Vis spectrophotometer |
|  | AgNP synthesis   1. Place the oil bath on the hot plate and set the temperature to 120 °C with stir on. 2. Weigh 147 mg of trisodium citrate and dissolve in 50 mL of ddiH_2_O to make 10 mM solution. 3. Weigh 42.5 mg of tannic acid and dissolve in 50 mL of ddiH_2_O to make 0.5 mM solution. 4. Mix the solutions prepared in step 2 and 3 in a cleaned round bottom flask to achieve final concentration of 5 mM of trisodium citrate and 0.25 mM of tannic acid, respectively. 5. Once the oil bath temperature reaches 120 °C, bring the flask and immerse it in the oil bath such that entire solution is immersed in the oil. 6. Bring the condenser to the mouth of the flask and switch on the water supply to avoid any water loss due to evaporation. 7. Let the solution to stir in oil bath for 15 minutes. 8. Once the solution starts to boil, remove the condenser, and add 1 mL of prepared silver nitrate solution (25 mM) in one shot using a micropipette. 9. An immediate change in color from colorless to yellow indicates the presence of nanoparticles. Continue to stir, with heat on, for 5 minutes. 10. Remove the flask from heating and allow it to stir at room temperature with condenser on until the solution has cooled to room temperature, about 45 minutes.   Purification of AgNPs   1. After the synthesized AgNPs have cooled down, transfer the contents to a centrifuge tube. 2. Centrifuge the sample for 15 minutes at 12000 rpm to remove unreacted reagents. 3. After centrifugation, discard the supernatant and collect the brown colored pellet. 4. Redisperse the pelleted nanoparticles in 10 mL of ddiH_2_O and repeat the centrifugation under the same conditions. 5. Repeat step 2-4 twice to wash the AgNPs and remove any unreacted materials. 6. Redisperse the AgNPs in 5 mL of ddiH_2_O.   Determine Concentration of AgNPs   1. Weigh an empty 1.5 mL microcentrifuge tube and record the mass. 2. Add 1 mL of previously prepared AgNP solution. 3. Centrifuge the tube and carefully remove the supernatant using a pipette without disturbing the pellet. 4. Vacuum dry the tube with the pellet and, using an analytical balance, measure the dried weight of the pellet, by subtracting the mass of the tube from the mass of the tube and the AgNP pellet. As this was performed using 1 mL of AgNP solution, the mass of the pellet is used as the mass concentration of AgNPs in the parent solution, in µg/mL.   UV-Vis characterization   1. Transfer 3 mL of the stored nanoparticles to a quartz cuvette. 2. Measure the absorbance from 200-800 nm for a 1 cm path length. 3. A single peak at 420 nm indicates the presence of silver nanoparticles. |
|  | **Sample Pre-Lab Questions** |
|  | 1. *Question:* What is the role of citrate in this reaction?   *Answer:* The citrate acts as the reducing agent and it covers the AgNPs after it is formed, increasing the stability of the AgNPs.   1. *Question:* How can we confirm that we have made AgNPs?   *Answer:* UV-Vis spectroscopy is a reliable means of assessing the formation of AgNPs.  ***Note to instructors:*** Other techniques are also acceptable here, such as atomic force microscopy, transmission electron microscopy, dynamic light scattering, nanodrop etc. |
|  | **Sample Post-Lab Questions** |
|  | 1. *Question:* Why does the solution change color once the AgNPs have formed?   *Answer:* AgNPs can absorb and scatter light in the visible spectrum*.*   1. *Question:* How would the UV-Vis spectrum change if we increased the size of the AgNPs? *Answer:* Increasing the size of the AgNPs would lead to them absorbing a longer wavelength of light*.* |

1. **DNA-Templated Silver Nanoclusters, DNA(C13)-AgNCs, Synthesis**

| **Timetable** |  |
| --- | --- |
| Preparation of Materials | 1 hour |
| DNA Resuspension | 15 – 30 minutes |
| Synthesis and Visualization | 1 hour |

| **Preparation of Materials** | |
| --- | --- |
|  | |
| **Buffers and Solutions**  *^*Prepare prior to the class session^* | |
|  | **Materials**   - ddiH_2_O CAS: 7732-18-5 - Ammonium acetate (NH_4­_OAc) powder CAS: 631-61-8 - Silver nitrate (AgNO_3_), 1 M solution CAS: 7761-88-8 - Glacial Acetic Acid (C_2_H_3_O_2_H) CAS: 64-19-7 - Sodium Hydroxide (NaOH), 2 M solution CAS: 1310-73-2   **Equipment**   - Precision balance - Weight boats - Scoopula - 1 L beaker - 50 mL sealable bottles or centrifuge tubes - 50 mL sealable bottles - pH meter - Micropipettes and pipette tips - Permanent marker - Parafilm - 1.5 mL centrifuge tubes - Vortexer - 500 mL graduated cylinder - Aluminum Foil |
|  | 20 mM Ammonium Acetate (NH_4_OAc) Buffer (pH 6.9) — 500 mL   1. Using a balance and scoopula, measure 0.77 g of NH_4_OAc into a weigh boat. 2. Transfer the NH_4_OAc into a clean, 1 L beaker and dissolve in 450 mL of ddiH_2_O. Use the solution to rinse any remaining NH_4_OAc from the weigh boat into the container. Vortex the solution until completely dissolved. 3. Using a calibrated pH meter, acetic acid, or sodium hydroxide, adjust the pH of the NH_4_OAc solution to 6.9. 4. Transfer the solution to a 500 mL graduated cylinder, rinsing the residual solution from the beaker into the cylinder. Dilute the solution to 500 mL with ddiH­_2_O. Transfer the solution to a labeled bottle. Store this solution at room temperature until use. Provide students with 1 mL aliquots in 1.5 mL centrifuge tubes.   10 mM Silver Nitrate (AgNO_3_) — 50 mL   1. Add 49.5 mL of ddiH_2_O to a 50 mL lidded container. 2. Add 500 µL of 1 M AgNO_3_ stock solution to the container. 3. Vortex the solution thoroughly. 4. Cover the container with aluminum foil and store at room temperature. Label the container. Provide students with 100 µL aliquots of this solution in 1.5 mL centrifuge tubes. |
| **DNA Resuspension**  *^*Prepare prior to the class session^* | |
|  | **Materials**   - DNA template (5’-TATCCGTCCCCCCCCCCCCCACGGATA-3’ from O’Neill et al.^1^) - ddiH_2_O CAS: 7732-18-5   **Equipment**   - Vortexer - Micropipettes and tips - Centrifuge |
|  | 1. Centrifuge the unopened tubes of lyophilized DNA template (purchased from IDT) to ensure none of the material is stuck in the cap from shipping. 2. Add an appropriate amount of ddiH_2_O, based on the number of nanomoles of DNA template were shipped, to make a 100 µM solution of DNA template. This can be easily accomplished by adding the number of microliters of ddiH_2_O equivalent ten times the number of nanomoles of DNA template provided. For example, if 100 nmol of DNA are received, adding 1,000 µL of ddiH_2_O yields a 100 µM solution of DNA template. 3. Vortex the solution thoroughly to ensure the DNA is fully dissolved. Store at 4 °C or on ice during use. Store at -20 °C for long-term storage. |
| **DNA(C13)-AgNC Synthesis and Visualization**  ^*Suggested to be done with students^ | |
|  | **Materials**   - 100 µM DNA-template solution - ddiH_2_O - Ice bath - 1.5 mL centrifuge tubes - 20 mM NH­_4_OAc Buffer (pH 6.9) - Sodium Borohydride (NaBH_4_) powder - Aluminum Foil - 10 mM AgNO­_3_ solution   **Equipment**   - Micropipettes and tips - Tube racks for 1.5 mL centrifuge tube - Vortexer - Centrifuge - Heatblock (95 °C) - Timer - Precision Balance - Scoopula - Stir rod - Weigh boat - Ultra-violet (UV) (260 ± 30 nm) light source - UV protective lab goggles - 150 mL beaker - 100 mL graduated cylinder - Parafilm |
|  | 1. Briefly vortex and centrifuge the DNA template and place it on ice. 2. Label 1.5 mL centrifuge tubes to read: “25 µM DNA(C13)-AgNCs” and “AgCtrl” (standing for the silver control sample). 3. The Supporting Table 1 below shows the calculations for synthesizing the DNA(C13)-AgNCs and AgCtrl samples. Note that all concentrations are shown in µM and volumes are given in µL. The volumes in blue are what students should add to each tube.   **Supporting Table 1.** The calculations for the synthesis of DNA(C13)-AgNCs and AgCtrl samples. The volumes in blue are added to each tube. For the AgCtrl sample, the DNA template volume is replaced with ddiH_2_O.   \| **DNA(C13)-AgNC Synthesis** \| \| **C1 (µM)** \| **V1 (µL)** \| **C2 (µM)** \| **ddiH_2_O (µL)** \| **V2 (µL)** \| \| --- \| --- \| --- \| --- \| --- \| --- \| --- \| \| #1 \| **C13 DNA Template** \| 100 \| 37.50 \| 25.00 \| 72.75 \| 150.00 \| \| **AgNO_3_** \| 10000.00 \| 4.88 \| 325.00 \| \| **20 mM NH_4_OAc** \| 20.00 \| 30.00 \| 4.00 \| \| #2 \| *vortex, centrifuge, 95°C/2min, 4°C/20min* \| \| \| \| \| \| #3 \| **NaBH_4_** \| 10000.00 \| 4.88 \| 325.00 \|  \|      \| **AgCtrl Synthesis** \| \| **C1 (µM)** \| **V1 (µL)** \| **C2 (µM)** \| **ddiH_2_O (µL)** \| **V2 (µL)** \| \| --- \| --- \| --- \| --- \| --- \| --- \| --- \| \| #1 \| **C13 DNA Template** \| 0 \| 0 \| 0 \| 110.25 \| 150.00 \| \| **AgNO_3_** \| 10000.00 \| 4.88 \| 325.00 \| \| **20 mM NH_4_OAc** \| 20.00 \| 30.00 \| 4.00 \| \| #2 \| *vortex, centrifuge, 95°C/2min, 4°C/20min* \| \| \| \| \| \| #3 \| **NaBH_4_** \| 10000.00 \| 4.88 \| 325.00 \|  \|  1. *Step #1:* Add 37.5 µL of DNA template solution, 4.88 µL of AgNO_3_, 30 µL of NH_4_OAc buffer and 72.75 µL of ddiH_2_O to the tube labeled, “25 µM DNA(C13)-AgNC.” To the tube labeled, “AgCtrl,” do not add any DNA template solution. 2. *Step #2:* Vortex and centrifuge the solutions briefly to ensure the solution is well mixed and collected at the bottom of the tube. Heat the solutions to 95 °C for 2 minutes. Quickly transfer the tubes from the heat block to an ice bath and allow them to cool for 20 minutes. 3. **Instructor Step:** During the 20 minutes incubation time, prepare a fresh 10 mM sodium borohydride solution. This should be done using cold ddiH_2_O and the solution should be kept in an ice bath until use. In order to determine the appropriate volume for the solution, measure between 0.01 — 0.03 g of NaBH_4_. Solve for the correct volume using Supporting Table 2 and Equation 1. The final volume, V, is a function of the measured mass of NaBH_4_, M, converted to the number of moles of NaBH_4_, n.   $V=\left( \frac{M}{37.832\frac{g}{mol}} \right)\left( \frac{1000 mL}{0.010 mol} \right)=n*\left( \frac{1000 mL}{0.010 mol} \right)$ (1)  **Supporting Table 2.** The table used to solve for the appropriate volume to dissolve a measured mass of NaBH_4_ in to achieve a 10 mM solution.   \|  \| **g/mol** \| **g** \| **mol** \| **ddiH2O (mL)** \| **C2 (mM)** \| \| --- \| --- \| --- \| --- \| --- \| --- \| \| NaBH4 \| 37.832 \| M \| n \| V \| 10.00 \|  1. In a graduated cylinder, measure the correct volume of cold ddiH_2_O, according to the measured mass of NaBH_4_. Add the NaBH_4_ to a beaker, submerged in an ice bath and rinse the weight boat into the beaker using 2-3 mL of ddiH_2_O. Add the remaining cold ddiH_2_O to the solution and thoroughly stir the solution to ensure it is well mixed. 2. *Step #3:* After the 20 minutes incubation in an ice bath or at 4 °C, add 4.88 µL of 10 mM NaBH_4_ solution to both the DNA(C13)-AgNCs and AgCtrl tubes. Gently mix the solutions with the pipet tip and return the tubes to the ice bath. 3. Place the samples in the dark and store them at 4 °C for 24 hours for the fluorescence to develop. 4. After the overnight development period, at least 8 hours, observe the red/orange fluoresce of the DNA(C13)-AgNC samples by illuminating them with 260 nm light. It may help to dim the lights in the laboratory during the visualization. The AgCtrl samples will not fluoresce. |
| **Sample Pre-Lab Questions** | |
|  | 1. What is the role of DNA in the synthesis of DNA-AgNCs?   *Answer: The DNA template acts as scaffold to bind the silver atoms in to the single-stranded cytosines. This allows the same DNA-AgNCs to be formed based on the structure and sequence of the DNA template.*   1. In addition to antibacterial uses, what other applications could there be for DNA-AgNCs?   *Answer: Due to their fluorescent properties, DNA-AgNCs have potential applications in nanomedicine for bioimaging and biosensing.*   1. What changes would be expected if a different DNA template were used?   *Answer: The fluorescence and antibacterial efficacy of the DNA-AgNC is based on the sequence/structure of the templating DNA strand. Changing the DNA template would likely result in a different color of fluorescence and different antibacterial properties.* |
| **Sample Post-Lab Questions** | |
|  | 1. *Question:* Why was the AgCtrl sample not fluorescent?   *Answer:* The AgCtrl sample did not have a DNA template to scaffold the formation of silver nanoclusters. As such, non-specific silver aggregates form in this solution instead of AgNCs.   1. *Question:* What and balance the chemical equation for the reduction of Ag^+^ by NaBH_4_.   *Answer:*  $2AgNO_{3}+2NaBH_{4}\to2Ag+2NaNO_{3}+B_{2}H_{6}+H_{2}$ |

1. **Antibacterial Efficacy Experiments**

| **Timetable** |  |
| --- | --- |
| Preparation of Materials | 4 hours |
| Treat and Plate Bacteria cultures | 3 hours |
| Count Colonies | 1 hour |

| **Preparation of Materials** | |
| --- | --- |
|  | |
| **Buffers and Solutions**  ^*Prepare prior to the class session^ | |
|  | **Materials**   - Lysogeny Broth (LB) dry powder - LB agar powder - Carbenicillin powder CAS: 4697-36-3 - ddiH_2_O CAS: 7732-18-5 - lyophilized K12 *E. coli* ATCC: ATCC 10798 - 100 mm x 25 mm petri dishes - Bleach solution (10 %) CAS: 7681-52-9 - Sterile pipette tips - Sterile 10 µL inoculation loop   **Equipment**   - Micropipettes - Autoclave - Balance - Scoopula - 1 L lidded bottles - Bunsen burner - Lighter - 1 L graduated cylinder - Sterile 50 mL centrifuge tubes - Autoclave indicator tape - Heat-resistant gloves - Autoclave - Parafilm - Biohazard waste container |
|  | Lysogeny Broth   1. Add 20 g of dry LB powder to a lidded bottle. 2. Measure 1 L of ddiH_2_O in a graduated cylinder and add it to the bottle with the LB powder. 3. Place the lid on the bottle loosely (never seal a container prior to autoclaving) and add a strip of autoclave indicator tape over the lid and side of the bottle. 4. Autoclave the bottle for 20 minutes at 121 °C. 5. Remove the bottle from the autoclave, using heat resistant gloves, and allow to cool to room temperature. 6. Aliquot the sterile LB into 20 aliquots of 50 mL in sterile centrifuge tubes. Seal the tubes and store them at 4 °C until use.   Pour LB Agar Plates   1. Add 20 g of dry LB powder to a lidded bottle. 2. Measure 1 L of ddiH_2_O in a graduated cylinder and add it to the bottle with the LB powder. 3. Place the lid on the bottle loosely (never seal a container prior to autoclaving) and add a strip of autoclave indicator tape over the lid and side of the bottle. 4. Autoclave the bottle for 20 minutes at 121 °C. 5. Remove the bottle from the autoclave, using heat resistant gloves, and allow to cool to about 60 °C. 6. While the bottle is cooling, sanitize your gloves and workspace with 10 % bleach solution. Dry the area with a paper towel. 7. Light a Bunsen burner and be sure to work near the flame to avoid any incidental contamination. *Note: An open flame should never be left unattended. It is always recommended to work with a partner when an open flame is in use.* 8. Open a new package of sterile petri dishes. Once the LB agar has reached about 60 °C, pour 20—25 mL into each petri dish (1 L of LB should make 40-50 petri dishes). 9. Allow the plates to cool and solidify under the Bunsen burner. 10. Once the plates are fully cooled, cover them with their lids, parafilm the outside and store them at 4 °C until use.   Carbenicillin Solution (5 mg/mL) in LB   1. Using a precision balance and a sterile scoopula, weigh out 125-250 mg of Carbenicillin powder onto a weigh boat. 2. Rinse the carbenicillin into a sterile 50 mL centrifuge tube with 3 mL of sterile LB. 3. Dilute the solution to the appropriate volume to make a 5 mg/mL solution. For example, if 125 mg of carbenicillin was measured, dissolve and dilute it to 25 mL with sterile LB.   Plate K12 *E. coli*   1. This initial plate will be used to pick individual colonies from for the student experiments. 2. Warm 1 mL of sterile LB to 37 °C. 3. Warm an LB agar plate to 37 °C. 4. Using a sterile scoopula, add a few grains of lyophilized K12 *E. coli* to the warm LB. 5. Incubate the culture at 37 °C with constant shaking at 200 rpm until it reaches an optical density of at least 0.2, measured at 600 nm. 6. Dip a sterile 10 µL inoculation loop into the culture and gently streak the plate using the pattern shown in Supporting Diagram 1 below.   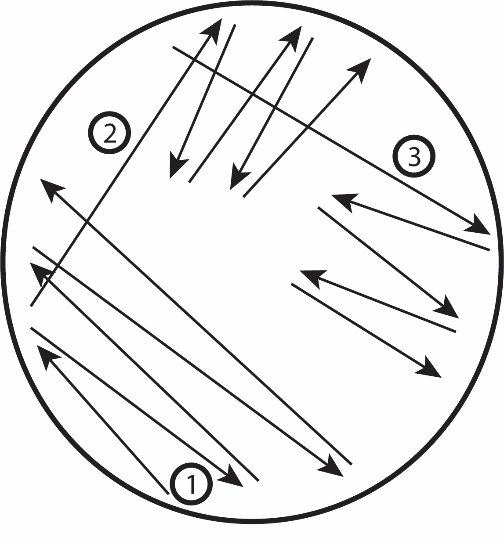  **Supporting Diagram 1.** This diagram shows how to streak the agar plate for your initial K12 culture. Starting at point 1, streak back and forth over a third of the plate, turn the plate and run the loop through the first streaks and then spread the culture over a small area (2). Do the same through area 3.   1. Cover the plate with its lid and incubate it at 37 °C upside down (with the agar on top) overnight. 2. The following morning, ensure that colonies have formed on the plate. Parafilm the outside of the plate and store at 4 °C until future use. |
| **Treat and Plate Bacteria Cultures**  ^*Suggested to be done with students^ | |
|  | **Materials**   - Sterile LB - Sterile phosphate buffered saline (PBS) pH 7.4 - DNA(C13)-AgNC solution—227.5 µg/mL (25 µM) - AgNP solution— between 250—600 µg/mL - NH_4_OAc buffer, 4 mM pH 6.9 - Carbenicillin solution in LB —5 mg/mL - LB agar plates - Sterile culture tubes with loose fitting lids - Test tube rack with magnetic bottom - 10% bleach solution - Paper towels   **Equipment**   - Bunsen burners - Lighter or strikers - Micropipettes and sterile tips - Shaking incubator (37 °C) - Incubator (37 °C) - Spectrophotometer - Biohazard waste container |
|  | Prepare Starter Culture—**Instructor Only**   1. The night before the class experiment, add 1 mL of sterile LB to a sterile culture tube. 2. Warm the LB to 37 °C. 3. Using a sterile 10 µL inoculation loop, pick 1 colony from the plated K12 *E. coli* and add it to the warm LB. 4. Incubate the starter culture overnight at 37 °C   Treat Bacterial Cultures   1. Place the LB-agar plates in a 37 °C incubator with the lids on the bottom (the agar face-down). Each student or group will need three plates. 2. Sterilize your gloves and working area by spraying with 10% bleach solution and wiping with a paper towel. 3. Label five sterile culture tubes with the each of the experimental conditions (Untreated Cells, DNA(C13)-AgNCs, AgNPs, Buffer, Carbenicillin). 4. Add each treatment with LB to the appropriately labeled tube according to Supporting Table 3. *Note: it is common for a color change to occur when AgNPs are added to LB.*   **Supporting Table 3.** This table shows the volumes to add of each treatment, LB, and dilute cell culture. Volumes in green are added to the tubes first to yield a 500 µL mixture of LB and treatment. The bacterial culture, 500 µL shown with a red background, is then added to reach the final volume of 1 mL.   \| **Treatment** \| **C_initial_ (ug/mL)** \| **V_added_ (µL)** \| **C_final_ (ug/mL)** \| **V_total_ (µL)** \| **LB_added_ (µL)** \| **Initial Culture (µL)** \| \| --- \| --- \| --- \| --- \| --- \| --- \| --- \| \| **DNA(C13)-AgNCs** \| 227.5 \| 132 \| 30 \| 1000 \| 368 \| 500 \| \| **AgNPs** \| 600 \| 50 \| 30 \| 1000 \| 450 \| 500 \| \| **Carbenicillin** \| 5000 \| 10 \| 50 \| 1000 \| 490 \| 500 \| \| **Buffer** \| **—** \| 132 \| **—** \| 1000 \| 368 \| 500 \| \| **Cells (untreated)** \| **—** \| 0 \| **—** \| 1000 \| 500 \| 500 \|  1. **Instructor Step**: While the students mix their treatments, blank a spectrophotometer with sterile LB at 600 nm. Dilute small amounts of the starter culture with sterile LB until it reaches a sufficient volume for the number of students (each student or group will use 2.5 mL of dilute culture) and an optical density, measured at 600 nm, between 0.015 and 0.02. 2. Once the treatments are mixed, students should add 500 µL of dilute culture (red background in table S3) to each of the tubes. 3. Incubate all of the student cultures at 37 °C, 200 rpm for 2 hours.   Dilute and Plate Bacterial Treatments  *Note: This should be started 30 minutes before the 2 hours incubation period has ended.*   1. Students should get twenty 1.5 mL centrifuge tubes, four per treatment, and label them with each treatments name and a dilution factor from 10^-1^—10^-4^. 2. Students should add 900 µL of sterile LB or PBS to each tube. 3. Once the incubation period is over, collect the culture tubes and LB agar plates, ensuring the plate remain face-down. 4. Label the LB agar plates according to Supporting Diagram 2 (below). Ensure that they are free of condensation on the agar surface.   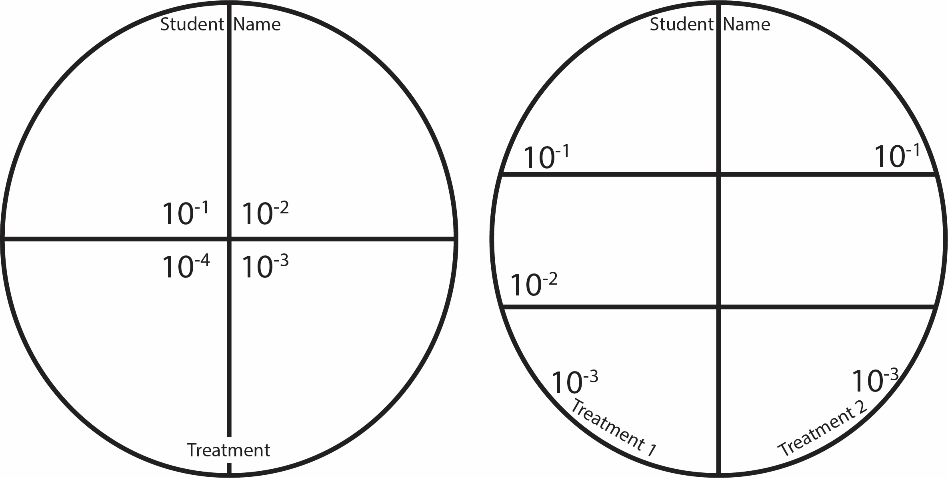  **Supporting Diagram 2.** The templates for how plates should be divided and labeled. Dilution factors can be adjusted as necessary. If there are enough plates for each student to use 1 plate per treatment, the left template, in which the plate is divided into quadrants should be used, with 20 µL of each dilution being placed in each quadrant. If fewer plates need to be used, two treatments can be plated on the same plate using the template on the right with 15 µL of each dilution being placed in each section. For the untreated cells, buffer treatment, and AgNP treatments, the dilution factors should include the range from 10^-2^–10^-4^. For the cultures treated with DNA(C13)-AgNCs or carbenicillin, the dilution factors should include the range from 10^-1^–10^-3^.   1. Light the Bunsen burner at your station. Work near the lit flame during when working with open bacteria cultures or LB agar plates. *Note: Be careful that no extremely loose-fitting clothing is worn near the Bunsen burner and that long hair is tied back to ensure everyone’s safety.* 2. Remove 100 µL from each treatment and add it to 900 µL of LB in that treatment’s 10^-1^ dilution. Mix this solution thoroughly by pipetting up and down. Change pipet tips and add 100 µL of the 10^-1^ dilution to 900 µL of LB in that treatment’s 10^-2^ dilution. Repeat this process to make the 10^-3^ and 10^-4^ dilutions for each sample. 3. Open the agar plates by keeping the lids on the benchtop and flipping the agar containing portion of the dish so the agar is face-up. 4. In the appropriate section of each plate, using sterile pipet tips, place three 5 µL drops (15 µL total) onto the agar, taking care not to touch the agar surface with your pipet tip. *Note: for AgNC and Carbenicillin samples, plate dilutions* 10^-1^–10^-3­^. *For all other samples, plate the* 10^-2^—10^-4^ *dilutions.* 5. Once the droplets are on the plate, take care not to disturb the plate, doing so may cause droplets to fuse across plate sectors, leading to less reliable results. Allow the droplets to dry by keeping the plate open near the lit flame of the Bunsen burner. 6. Once the droplets have dried on the plate’s surface, close the plates by flipping them face-down onto their lids. Turn off the Bunsen burner. Transfer the plates to a 37 °C incubator. 7. Incubate the plates overnight at 37 °C. 8. Sanitize your workstation and equipment by spraying everything with 10% bleach solution and wiping it off with a paper towel. 9. Dispose of all waste in the biohazard waste container.   Count Colonies on Agar Plates   1. Retrieve the agar plates from the incubator and place them at your workbench face-down. 2. Using a fine point permanent marker, place a dot over each colony as you count them. 3. Record the total number of counted colonies for each dilution in a table. 4. Dispose of the plates in the biohazard waste container. 5. Sanitize your workspace by spraying it with 10% bleach solution and wiping it with a paper towel. |
| **Sample Pre-Lab Questions** | |
|  | 1. *Question:* Which sample has more silver in a single particle, AgNPs or DNA(C13)-AgNCs? *Answer:* AgNPs have more silver per particle as they are ~ 50 nm spheres of mostly silver atoms while DNA(C13)-AgNCs have about 10 silver atoms bound to the DNA template. 2. *Question:* What does it mean for a strain of bacteria to be non-pathogenic?   *Answer:* It means that this strain of bacteria is generally not capable of infecting someone. Despite this, it should still be handled safely and with proper laboratory protocol.   1. *Question:* What safety precautions should be taken around a lit Bunsen burner?   *Answer:* Loose-fitting clothing should not be worn near a Bunsen burner and long hair should be tied back. Additionally, a lit flame should never be left unattended. |
| **Sample Post-Lab Questions** | |
|  | 1. *Question:* From looking at the liquid cultures, can you guess which treatment was the most effective and which was the least effective?   *Answer:* The carbenicillin and DNA(C13)-AgNC samples should appear to be much less turbid than the other samples, which should look similarly turbid.   1. *Question:* Calculate the number of colony forming units per milliliter of culture (CFU/mL) from your colony counting results.   *Answer:* Use equation 1 in the main text.   1. *Question:* Which sample was the most effective and which was the least effective?   *Answer:* DNA(C13)-AgNCs and carbenicillin should be the most effective when compared to the AgNPs, Buffer, and untreated cells*.* |

1. **Reagent Cost Estimate**

To estimate the cost per student for the reagents and materials, prices were gathered from a variety of scientific suppliers and the best price/unit was used. This process did not account for any bulk order, instrumental costs, or institutional discounts.

For a single group to complete the entire series of experiments, it would cost $6.81. It is recommended that students work in groups of either two ($3.41/student) or three ($2.27/student).

For a group of 25 students working in 12 groups, it is sufficient to order the DNA at a 250 nmol synthesis scale with standard desalting and no further purification. It is recommended to order the oligonucleotide in its lyophilized form and resuspend it in ddiH_2_0 upon arrival. It should be stored frozen until use.

1. **References**

1. O’Neill, P. R.; Velazquez, L. R.; Dunn, D. G.; Gwinn, E. G.; Fygenson, D. K., Hairpins with Poly-C Loops Stabilize Four Types of Fluorescent Agn:DNA. *The Journal of Physical Chemistry C* **2009,** *113* (11), 4229-4233.
