# Supplementary material for: Demonstrating the Synthesis and Antibacterial Properties of Nanostructured Silver
Source: J Chem Educ. Author manuscript; Available in PMC 2023 Sep 19. (PMC10501122; doi:10.1021/acs.jchemed.3c00125)
Supplement: SI2 [file NIHMS1930025-supplement-SI2.docx]

Supporting Information

**Demonstrating the Synthesis and Antibacterial Properties of Nanostructured Silver**

Lewis Rolband^1#^, Varsha Godakhindi^1#^, Juan L. Vivero-Escoto^1*^, Kirill Afonin^1*^

^1^Department of Chemistry, University of North Carolina at Charlotte, Charlotte, North Carolina 28223, United States

^#^L.R. and V.G. contributed equally

^*^Corresponding Authors: [kafonin@uncc.edu](mailto:kafonin@uncc.edu); juan.vivero-escoto@uncc.edu


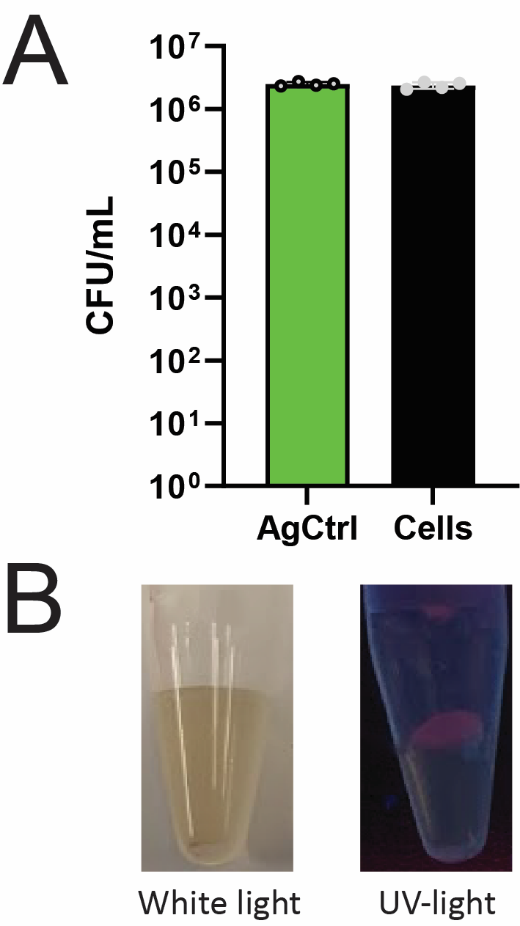


**Figure S1.** AgCtrl samples, prepared as described in the main text were used to treat K12 *E. coli*, using an equivalent volume of AgCtrl solution as was used for DNA(C13)-AgNC treatments. (A) The results of the colony counting are shown to compared against untreated K12 *E. coli*, demonstrating that the reduced Ag, lacking the DNA-template, is ineffective as an antibacterial agent. (B) Representative photographs of the AgCtrl solution are shown under illumination with white light and transillumination with 254 nm light.


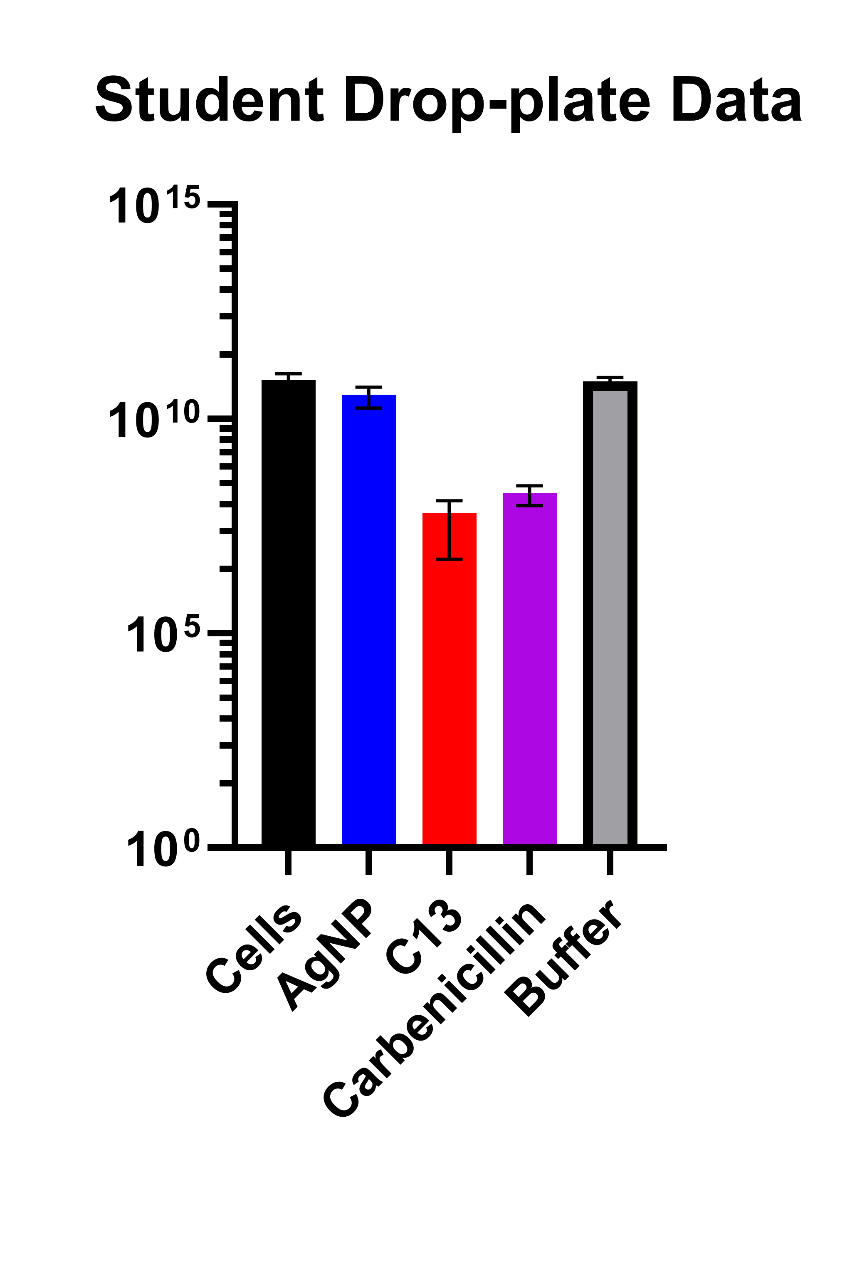


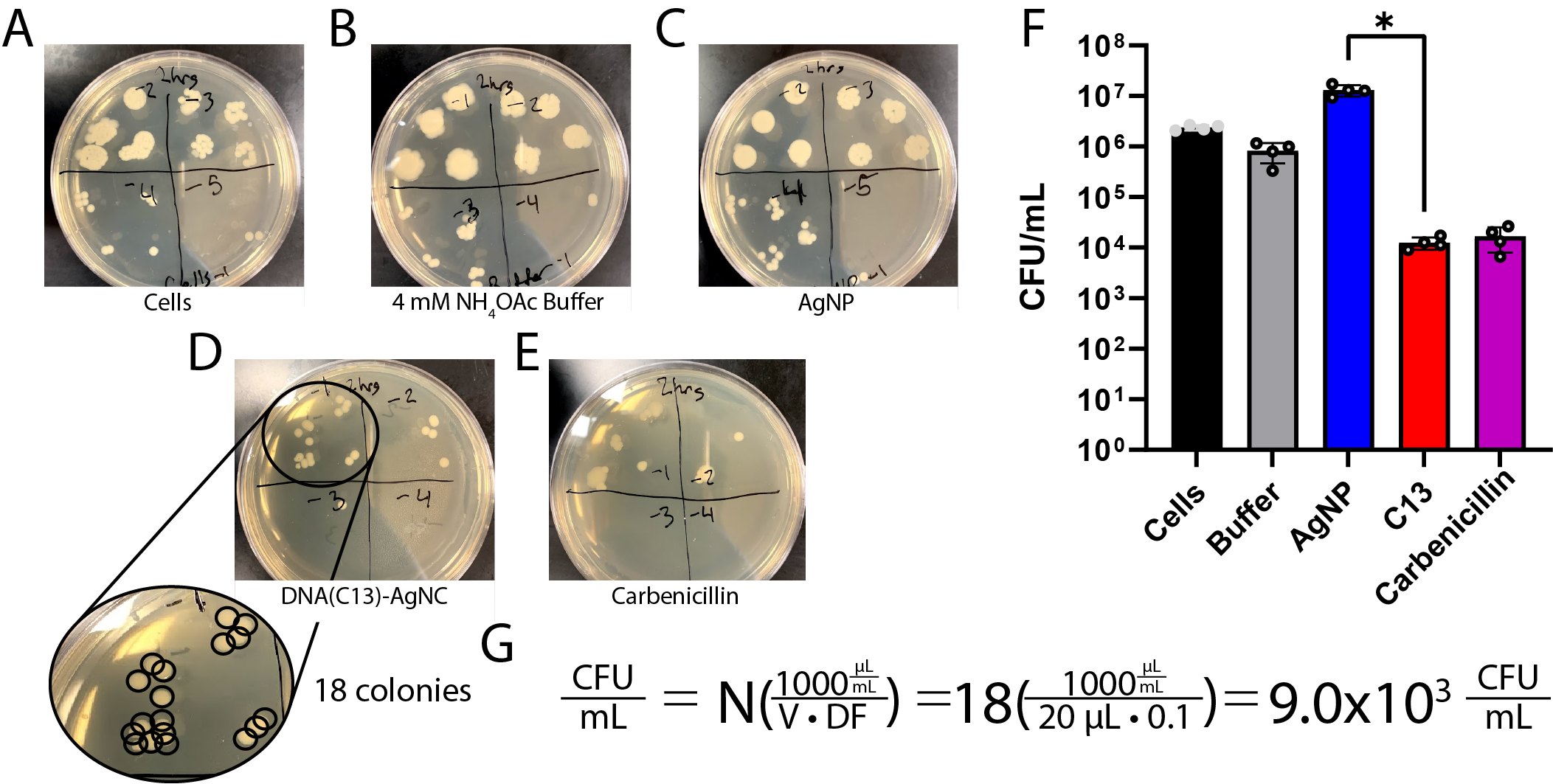
**Figure S2.** The averaged data from five groups of students, working in groups of two or three individuals, is shown. The trends seen in the instructor’s representative data, Figure 3 in the main text, are clearly seen in the student data. The students have higher numbers bacteria in each culture, however. This is due to the increased length of time it took the students to prepare each treatment solution, as the freshly diluted bacteria stock solution continuing to grow during the delay, resulting in the students beginning the experiment with a higher concentration of bacteria in their initial solutions.
