# Supplementary material for: Demonstrating the Synthesis and Antibacterial Properties of Nanostructured Silver
Source: J Chem Educ. Author manuscript; Available in PMC 2023 Sep 19. (PMC10501122; doi:10.1021/acs.jchemed.3c00125)
Supplement: SI6 [file NIHMS1930025-supplement-SI6.docx]

Supporting Information

**Demonstrating the Synthesis and Antibacterial Properties of Nanostructured Silver**

Lewis Rolband^1#^, Varsha Godakhindi^1#^, Juan L. Vivero-Escoto^1*^, Kirill Afonin^1*^

^1^Department of Chemistry, University of North Carolina at Charlotte, Charlotte, North Carolina 28223, United States

^#^L.R. and V.G. contributed equally

^*^Corresponding Authors: kafonin@uncc.edu; juan.vivero-escoto@uncc.edu

**AgNP Synthesis and Characterization**

**Materials**

- Double-deionized water (ddiH_2_O, 18 mΩ)
- Trisodium citrate
- Tannic acid
- Silver Nitrate (AgNO_3_), 25 mM
- Quartz cuvette
- Centrifuge tube (50 mL)

**Equipment**

- Cleaned Round bottom flask (250 mL)
- Cleaned Stir bar
- Beakers
- Graduated cylinder
- Oil bath
- Hot plate with stirrer
- Temperature probe
- Condenser
- Precision balance
- Weighing paper
- Scoopula
- Micropipette and pipette tips (1000 µL)
- Sharpie marker
- Glass vial (20 mL)
- Centrifuge
- UV-Vis spectrophotometer

**Silver Nanoparticle (AgNP) synthesis**

1. Place the oil bath on the hot plate and set the temperature to 120 °C with stir on.
2. Weigh 147 mg of trisodium citrate and dissolve in 50 mL of ddiH_2_O to make 10 mM solution.
3. Weigh 42.5 mg of tannic acid and dissolve in 50 mL of ddiH_2_O to make 0.5 mM solution.
4. Mix the solutions prepared in step 2 and 3 in a cleaned round bottom flask to achieve final concentration of 5 mM of trisodium citrate and 0.25 mM of tannic acid.
5. Once the oil bath temperature reaches 120 °C, bring the flask and immerse it in the oil bath such that entire solution is immersed in the oil.
6. Bring the condenser to the mouth of the flask and switch on the water supply to avoid any water loss due to evaporation.
7. Let the solution to stir in oil bath for 15 minutes.
8. Once the solution starts to boil, remove the condenser, and add 1 mL of prepared silver nitrate solution (25 mM) in one shot using a micropipette.
9. An immediate change in color from colorless to yellow indicates the presence of nanoparticles. Continue to stir, with heat on, for 5 minutes.
10. Remove the flask from heating and allow it to stir at room temperature with condenser on until the solution has cooled to room temperature, about 45 minutes.

**Purification of AgNPs**

1. After the synthesized AgNPs have cooled down, transfer the contents to a centrifuge tube.
2. Centrifuge the sample for 15 minutes at 12000 rpm to remove unreacted reagents.
3. After centrifugation, discard the supernatant and collect the brown colored pellet.
4. Redisperse the pelleted nanoparticles in 10 mL of ddiH_2_O and repeat the centrifugation under the same conditions.
5. Repeat step 2-4 twice to wash the AgNPs and remove any unreacted materials.
6. Redisperse the AgNPs in 5 mL of ddiH_2_O.

**Determine Concentration of AgNPs**

1. Weigh an empty 1.5 mL microcentrifuge tube and record the mass.
2. Add 1 mL of previously prepared AgNP solution.
3. Centrifuge the tube and carefully remove the supernatant using a pipette without disturbing the pellet.
4. Vacuum dry the tube with the pellet and, using an analytical balance, measure the dried weight of the pellet, by subtracting the mass of the tube from the mass of the tube and the AgNP pellet. As this was performed using 1 mL of AgNP solution, the mass of the pellet is used as the mass concentration of AgNPs in the parent solution, in µg/mL.

**UV-Vis characterization**

1. Transfer 3 mL of the stored nanoparticles to a quartz cuvette.
2. Measure the absorbance from 200-800 nm for a 1 cm path length.
3. A single peak at 420 nm indicates the presence of silver nanoparticles.

**DNA(C13)-AgNC Synthesis and Visualization**

**Materials**

- 100 µM DNA(C13)-template solution
- ddiH_2_O Ice bath
- 1.5 mL centrifuge tubes
- 20 mM NH­_4_OAc Buffer (pH 6.9)
- Sodium Borohydride (NaBH_4_) powder
- Aluminum Foil
- 10 mM AgNO­_3_ solution

**Equipment**

- Micropipettes and tips
- Tube racks for 1.5 mL centrifuge tube
- Vortexer
- Centrifuge
- Heatblock (95 °C)
- Timer
- Precision Balance
- Scoopula
- Stir rod
- Weigh boat
- Ultra-violet (UV) light source
- UV protective lab goggles
- 150 mL beaker
- 100 mL graduated cylinder
- Parafilm

**DNA(C13)-AgNC Synthesis**

1. Briefly vortex and centrifuge the DNA template and place it on ice.
2. Label 1.5 mL centrifuge tubes to read: “25 µM DNA(C13)-AgNCs” and “AgCtrl” (standing for the silver control sample)
3. The Supporting Table 1 below shows the calculations for synthesizing the DNA(C13)-AgNCs and AgCtrl samples. Note that all concentrations are shown in µM and volumes are given in µL. The volumes in blue are what students should add to each tube.

**Supporting Table 1.** The calculations for the synthesis of AgNCs and AgCtrl samples. The volumes in blue are added to each tube. For the AgCtrl sample, the DNA template volume is replaced with ddiH_2_O.

| *Step* | **DNA(C13)-AgNC Synthesis** | **C1 (µM)** | **V1 (µL)** | **C2 (µM)** | **ddiH_2_O (µL)** | **V2 (µL)** |
| --- | --- | --- | --- | --- | --- | --- |
| *#1* | **C13 DNA Template** | 100 | 37.50 | 25.00 | 72.75 | 150.00 |
|  | **AgNO_3_** | 10000.00 | 4.88 | 325.00 |  |  |
|  | **20 mM NH_4_OAc** | 20.00 | 30.00 | 4.00 |  |  |
| *#2* | *vortex, centrifuge, 95°C/2min, 4°C/20min* | | | | |  |
| *#3* | **NaBH_4_** | 10000.00 | 4.88 | 325.00 |  |  |

| *Step* | **AgCtrl Synthesis** | **C1 (µM)** | **V1 (µL)** | **C2 (µM)** | **ddiH_2_O (µL)** | **V2 (µL)** |
| --- | --- | --- | --- | --- | --- | --- |
| #1 | **C13 DNA Template** | 0 | 0 | 0 | 110.25 | 150.00 |
|  | **AgNO_3_** | 10000.00 | 4.88 | 325.00 |  |  |
|  | **20 mM NH_4_OAc** | 20.00 | 30.00 | 4.00 |  |  |
| #2 | *vortex, centrifuge, 95°C/2min, 4°C/20min* | | | | |  |
| #3 | **NaBH_4_** | 10000.00 | 4.88 | 325.00 |  |  |

1. *Step #1:* Add 37.5 µL of DNA template solution, 4.88 µL of AgNO_3_, 30 µL of NH_4_OAc buffer and 72.75 µL of ddiH_2_O to the tube labeled, “25 µM DNA(C13)-AgNC.” To the tube labeled, “AgCtrl,” do not add any DNA template solution.
2. *Step #2:* Vortex and centrifuge the solutions briefly to ensure the solution is well mixed and collected at the bottom of the tube. Heat the solutions to 95 °C for 2 minutes. Quickly transfer the tubes from the heat block to an ice bath and allow them to cool for 20 minutes.
3. *Step #3:* After the 20 minutes incubation period in an ice bath or at 4 °C, add 4.88 µL of 10 mM NaBH_4_ solution to both the DNA(C13)-AgNCs and AgCtrl tubes. Gently mix the solutions with the pipet tip and return the tubes to the ice bath.
4. Place the samples in the dark and store them at 4 °C for 24 hours for the fluorescence to develop.
5. After the 24 hours development period, observe the red/orange fluoresce of the DNA(C13)-AgNC samples by illuminating them with UV light. It may help to dim the lights in the laboratory during the visualization. The AgCtrl samples will not fluoresce.

**Treat and Plate Bacteria Cultures**

**Materials**

- Sterile LB
- DNA(C13)-AgNC solution—227.5 µg/mL (25 µM)
- AgNP solution— between 250—600 µg/mL
- NH_4_OAc buffer, 4 mM pH 6.9
- Carbenicillin solution in LB —5 mg/mL
- LB agar plates
- Sterile culture tubes with loose fitting lids
- Test tube rack with magnetic bottom
- 10% bleach solution
- Paper towels

**Equipment**

- Bunsen burners
- Lighter or strikers
- Micropipettes and sterile tips
- Shaking incubator (37 °C)
- Incubator (37 °C)
- Spectrophotometer
- Biohazard waste container

**Treat Bacterial Cultures**

1. Place the LB-agar plates in a 37 °C incubator with the lids on the bottom (the agar face-down). Each student or group will need three plates.
2. Sterilize your gloves and working area by spraying with 10% bleach solution and wiping with a paper towel.
3. Label five sterile culture tubes with the each of the experimental conditions (Untreated Cells, DNA(C13)-AgNCs, AgNPs, Buffer, Carbenicillin).
4. Add each treatment with LB to the appropriately labeled tube according to Supporting Table 2 (below). *Note: it is common for a color change to occur when AgNPs are added to LB.*

**Supporting Table 2.** This table shows the volumes to add of each treatment, LB, and dilute cell culture. Volumes in green are added to the tubes first to yield a 500 µL mixture of LB and treatment. The bacterial culture, 500 µL shown with a red background, is then added to reach the final volume of 1 mL.

| **Treatment** | **C_initial_ (ug/mL)** | **V_added_ (µL)** | **C_final_ (ug/mL)** | **V_total_ (µL)** | **LB_added_ (µL)** | **Initial Culture (µL)** |
| --- | --- | --- | --- | --- | --- | --- |
| **DNA(C13)-AgNCs** | 227.5 | 132 | 30 | 1000 | 368 | 500 |
| **AgNPs** | 600 | 50 | 30 | 1000 | 450 | 500 |
| **Carbenicillin** | 5000 | 10 | 50 | 1000 | 490 | 500 |
| **Buffer** | **—** | 132 | **—** | 1000 | 368 | 500 |
| **Cells (untreated)** | **—** | 0 | **—** | 1000 | 500 | 500 |

1. Once the treatments are mixed, students should add 500 µL of dilute culture (red background in table S3) to each of the tubes.
2. Incubate all of the student cultures at 37 °C, 200 rpm for 2 hours.

**Dilute and Plate Bacterial Treatments**

*Note: This should be started 30 minutes before the 2 hours incubation period has ended.*

1. Students should get twenty 1.5 mL centrifuge tubes, four per treatment, and label them with each treatments name and a dilution factor from 10^-1^—10^-4^.
2. Students should add 900 µL of sterile LB or phosphate buffered saline (PBS) to each tube.
3. Once the incubation period is over, collect the culture tubes and LB agar plates, ensuring the plate remain face-down.
4. Label the LB agar plates according to Supporting Figure 1. Ensure that they are free of condensation on the agar surface.


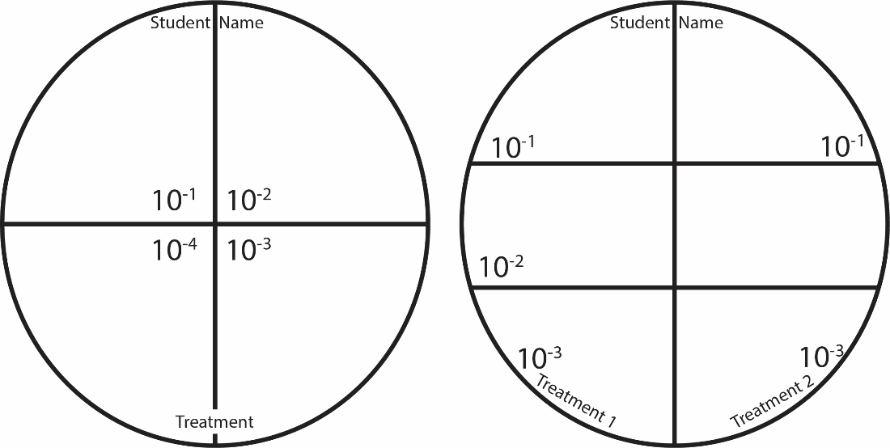


**Supporting Figure 1.** The template for how plates should be divided and labeled. If using one plate per treatment, use the template on the left. If using one plate for two treatments, use the template on the right. Dilution factors can be adjusted as necessary. For the untreated cells, buffer treatment, and AgNP treatments, the dilution factors should include the range from 10^-2^–10^-4^. For the cultures treated with DNA(C13)-AgNCs or carbenicillin, the dilution factors should include the range from 10^-1^–10^-3^.

1. Light the Bunsen burner at your station. Work near the lit flame during when working with open bacteria cultures or LB agar plates. *Note: Be careful that no extremely loose-fitting clothing is worn near the Bunsen burner and that long hair is tied back to ensure everyone’s safety.*
2. Remove 100 µL from each treatment and add it to 900 µL of LB/PBS in that treatment’s 10^-1^ dilution. Mix this solution thoroughly by pipetting up and down. Change pipet tips and add 100 µL of the 10^-1^ dilution to 900 µL of LB in that treatment’s 10^-2^ dilution. Repeat this process to make the 10^-3^ and 10^-4^ dilutions for each sample.
3. Open the agar plates by keeping the lids on the benchtop and flipping the agar containing portion of the dish so the agar is face-up.
4. In the appropriate section of each plate, using sterile pipet tips, place three 5 µL drops (15 µL total) onto the agar, taking care not to touch the agar surface with your pipet tip. *Note: for AgNC and Carbenicillin samples, plate dilutions* 10^-1^—10^-3­^. *For all other samples, plate the* 10^-2^—10^-4^ *dilutions.*
5. Once the droplets are on the plate, take care not to disturb the plate, doing so may cause droplets to fuse across plate sectors, leading to less reliable results. Allow the droplets to dry by keeping the plate open near the lit flame of the Bunsen burner.
6. Once the droplets have dried on the plate’s surface, close the plates by flipping them face-down onto their lids. Turn off the Bunsen burner. Transfer the plates to a 37 °C incubator.
7. Incubate the plates overnight at 37 °C.
8. Sanitize your workstation and equipment by spraying everything with 10% bleach solution and wiping it off with a paper towel.
9. Dispose of all waste in the biohazard waste container.

**Count Colonies on Agar Plates**

1. Retrieve the agar plates from the incubator and place them at your workbench face-down.
2. Using a fine point permanent marker, place a dot over each colony as you count them.
3. Record the total number of counted colonies for each dilution in a table.
4. Dispose of the plates in the biohazard waste container.
5. Sanitize your workspace by spraying it with 10% bleach solution and wiping it with a paper towel.
